# ‘Standing on the Shoulders of the Giants’: Dr. Robert Kyle

**DOI:** 10.1007/s44228-022-00021-7

**Published:** 2022-11-08

**Authors:** Mohamad Mohty, Robert A. Kyle

**Affiliations:** 1 Sorbonne Université, AP-HP, INSERM UMRs938, Paris, France; 2 Service d’Hématologie Clinique et de Thérapie Cellulaire, Hôpital Saint Antoine, AP-HP, Paris, France; 3 Division of Hematology, Mayo Clinic, Rochester, MN USA

**Keywords:** Multiple myeloma, Monoclonal gammopathy

In a letter to Robert Hooke in 1675, Sir Isaac Newton made his most famous statement: *“if I have seen further, it is by standing on the shoulders of giants.”*

**Fig. 1:**
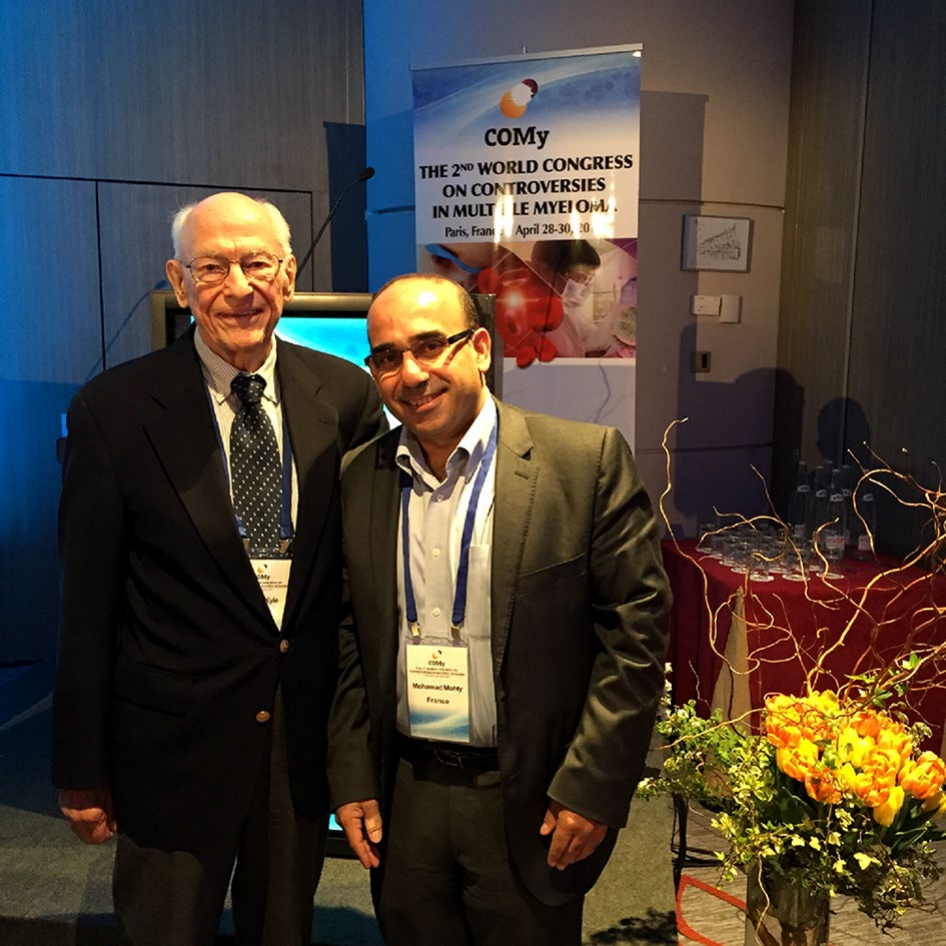
Professor Kyle and Professor Mohty in 2016 at the 2nd International Congress for Controversies in Multiple Myeloma (COMy 2016)

This initiative of the International Academy for Clinical Hematology (IACH) aims to celebrate the achievements of leading experts and investigators whose work and research have helped to significantly advance the field of clinical hematology and establish the milestones and foundations of modern clinical hematology. The IACH Scientific Steering Committee will choose the honorees from different fields of clinical hematology. The choice is based on the nominee’s body of work, including strength of research, clinical impact, significant educational contributions, and overall accomplishments. This report represents a transcript of the interview given by Dr. Robert A. Kyle (RK) on April 12, 2022, who responded to a series of questions asked by Dr. Mohamad Mohty (MM; Fig. 1).

**MM:** Our honoree today is Professor Robert A. Kyle, who is a living legend in medicine. Robert A. Kyle, MD is Professor of Medicine, Laboratory Medicine, and Pathology at the Mayo Clinic College of Medicine, in Rochester, Minnesota. The scientific contributions of Dr. Kyle over a remarkable 60-year career in medicine have no parallel, which is why we are very proud to have you with us, Dr. Kyle, today. So, Dr. Kyle, before going into your scientific achievements and answering the many questions I have for you, I wonder if you can tell us something about your early childhood and early educational experience, and how they may have played a role in the selection of your scientific and medical fields.

**RK:** Good morning, Mohamad, thank you very much for those very kind words.

I was born in North Dakota, a state that borders Canada, in the middle of the United States. My father ran a tire repair shop, but after several years, decided that this was not a very healthy occupation so he bought a farm and moved there near our hometown of Bottineau in 1930. I went to a one-room country school that had eight grades, in which one had to take state examinations in order to proceed to high school. During this time, my mother was visiting a neighbor and told her that I was a very good student but that she didn’t know what I was going to do. The neighbor said why didn’t I become a doctor. This was the very first seed of the idea, which sounded good to me. I enjoyed school and was doing well, and I would rather have continued studying than go to work. I carried on and after high school went to a junior college called the North Dakota School of Forestry, in Bottineau. I entered the pre-med program and not forestry. However, I did work as a Lookout-Fireman for two summers, an enjoyable job but not my future interest!

I continued in pre-medical studies and graduated from the University of North Dakota in 1948. The Dean of the Medical School interviewed me at the North Dakota School of Medicine at Grand Forks which had a 2-year program and said that he would accept me there but that it would be better to go to a 4-year medical school. He told me that if I wanted to be a Professor, I should go to Harvard; if I wanted to do research, I should go to the University of Pennsylvania or Johns Hopkins University; but if I wanted to be a ‘real doctor,’ I should attend Northwestern University Medical School (NUMS) in Chicago. At that time, obviously I wanted to be a ‘real doctor’ and so I applied to NUMS and was accepted. I enjoyed all aspects of the courses but decided to focus on internal medicine and took my internship at a university hospital in Chicago, which emphasized internal medicine. I then applied to the Mayo Clinic as I had been told that it had the best internal medicine program.

At that time, I want to emphasize that internal medicine was considered a specialty such that after 3 years’ training, one would take the board exams and become a practicing internist. I began my training in Rochester but after a year, was drafted into the U.S. Air Force and spent 2 years in Alaska. I was in a 400-bed hospital and ended up in charge of the major medical ward. I saw a lot of interesting diseases, and more importantly, I was responsible and had to manage a lot of things and consequently learned a lot. I returned to the Mayo Clinic, and during the last year of my fellowship, I took the hospital service on hematology where the consultant showed me an electrophoretic pattern. I asked what it meant, to which Dr. E.D. Bayrd replied that it was a new test and “why don’t you look into it.” I reviewed 3 years of output of electrophoretic patterns and realized that patients with multiple myeloma and Waldenström macroglobulinemia had an abnormal protein spike. I defined the criterion of these abnormal spikes as having a height/width ratio of 4:1 or greater.

**MM:** So, since your first publication in 1959, you have defined and redefined the epidemiology, diagnosis, risk stratification, prognosis, and management of the entire spectrum of monoclonal gammopathy of undetermined significance. This is fascinating, from a one-room school to the Mayo Clinic reviewing the electrophoresis patterns. Was there a particular teacher or faculty that you thought at that time to be exemplary of the academic tradition and that you wanted to follow on the same path?

**RK:** Well, the most influential person was a specialist in multiple myeloma, Dr. Ned Bayrd. He was the one who told me to “look into it” when I asked about the electrophoretic patterns and that led me to reviewing them all. On that same hospital service, there was a patient thought to have multiple myeloma who, when admitted, did not have holes in her bones or severe anemia and other features of active myeloma. She had changes in her skin and had a skin biopsy, which was reported as amyloidosis. Amyloidosis was something that we had seen in pathology. Despite my three and a half years at the Mayo Clinic in internal medicine and 2 years of practice in Alaska, this was the first time that I had recognized this disease. I then reviewed all of the cases of amyloidosis at the Mayo Clinic and described primary systemic amyloidosis. This was the start of my studies in the monoclonal gammopathies, which has kept me occupied for the past 60 years.

**MM:** This is amazing. You started this story by reviewing all of the electrophoresis patterns. Can you talk about the structure of the hematology field back in the 60s when you started your research in this area?

**RK:** Actually, in the 1960s I was involved with the national cooperative group of Acute Leukemia Group B, and I saw a lot of acute myelocytic leukemia (AML). I became curious and looked at the histories of the previous 50 patients with AML, and found that they had a very short survival. This was an eye-opening experience, which exposed me to the fact that there was “no useful treatment” for AML at that time. Melphalan had been used in the late 1950s in Russia and then by Professor David A.G. Galton in London, and finally, in the United States. I would emphasize that things didn’t move nearly as quickly, from a scientific standpoint, as they do today.

**MM:** Can you tell us about the sort of research and clinical problems that people were debating in the field of blood and bone marrow diseases when your interest in monoclonal gammopathies began?

**RK:** One of the major areas of interest was the radioisotopes, and I was told in medical school during the one-hour course in 1949 that it was the first course on radioisotopes for medical students in the United States. Radioactive iron was used to study blood loss for example, but I never really became involved with radioisotopes from a research standpoint but was occupied with the serum protein electrophoretic patterns.

**MM:** So, are there any people who were active in research at that time that you would like to talk about that had a particular influence on you and your research?

**RK:** Well, the Head of Clinical Pathology, Chuck Owen, who had trained at the University of Iowa, was a fantastic teacher. For example, he was particularly interested in the field of coagulation, which was always a confusing area, but after a one-hour lecture by Chuck, it was all very clear. However, within a few days, things clouded over, and I was no wiser than I was before. It was a frustrating area as far as I was concerned. I wasn’t the only one though; most of the students and young consultants felt the same, unless they were involved in coagulation practice or research.

**MM:** So, coagulation was definitely not your cup of tea! But could you go a bit further into the intellectual interactions that took place at the Mayo Clinic when you started this new field that you have, I would say, almost invented.

**RK:** When I began at the Mayo Clinic, the educational program consisted of a series of lectures on Monday and Thursday evenings. We were told that if we attended these, we would pass the American Board of Internal Medicine without any problem whatsoever. I want to emphasize that internal medicine was a specialty choice, like surgery, in which I had no interest. On Tuesday evenings we had seminars in medicine for a year and then in our second year we had seminars in physiology. In both of these, a fellow gave one of these presentations during the academic year so one spent a lot of time in preparation. At the end of the year, the presentations were published. This gave one very good exposure to internal medicine. When it came to physiology and the time to select my research project, I really wasn’t interested in the intricacies of physiology because at that time, open-heart surgery had just started at the Mayo Clinic and at the University of Minnesota. I don’t think it has ever been resolved who the first one was, as it depended on who you listened to. Cardiac catheterization was a large part of the curriculum. A fellow could give his seminar on pathology but it didn’t look to me as if pathology would be very interesting for 6 months. One had to select a project and write a thesis, which would subsequently be examined by the University of Minnesota Graduate School. One had to go to Minneapolis and be questioned by the Graduate Committee of the University, which was a rather traumatic experience. I was fortunate in that I was asked questions that I happened to know the answers. In order to get that Master’s degree, one also had to write the thesis, defend it, and then write and publish the paper in a medical journal. Mine was on hemolytic anemia, and that’s how I got started in hematology. It was after that that I took that hospital service and was introduced to multiple myeloma and amyloidosis, as I have already mentioned.

**MM:** You have mentioned fellows and students and myeloma, and you have trained dozens of myeloma doctors at the Mayo Clinic, in the United States, and across the globe. Did you see yourself as playing the role of influencing physicians in any way, as you moved towards this field of myeloma and gammopathies in general?

**RK:** Yes, I wanted to interest them in the field as there were not many physicians with a lot of experience or interest in multiple myeloma. You can imagine that at that time, most people were general internists and as you well know, hematology accounts for only a modest number of patients, compared with cardiology, gastroenterology, or pulmonary diseases. When a fellow left here and went into private practice and saw a patient with multiple myeloma, they often would telephone me and ask for advice. I took a lot of those calls, particularly about patients with amyloidosis because no one had had much experience with it.

**MM:** I think this brings me to another key question. Your work has made a difference to thousands and thousands of patients worldwide, which is why I am very interested in your relationship with patients and how, looking at particular disease symptoms, this has influenced the sort of questions that you have chosen to ask over the last 60 years?

**RK:** Well, I have always said that patients have taught me everything that I know! For example, the idea of monoclonal gammopathy of undetermined significance (MGUS) first arose when I was reviewing a patient’s history while looking at Mayo Clinic patients with multiple myeloma in the 1960s. I collected all of the patients and that is the paper I published emphasizing the serum protein electrophoretic patterns of those individuals. The most important patient in this series was one with multiple myeloma who had been to the Mayo Clinic in 1945 complaining of weakness and fatigue (a complaint of many patients) and the hematologist who saw her ordered an albumin/globulin ratio (serum protein electrophoresis was not yet available), which had been done since the 1920s. The globulin level was elevated, the patient’s sedimentation rate was > 100 mm/h, but the patient looked well and the rest of her laboratory tests were normal and wisely, the internist who saw her did not treat her. She returned about 15 years later, at which time electrophoresis was available and her pattern showed a spike of 2.9 g/dl. I had chosen 3.0 g/dl as the cutoff, above which indicated multiple myeloma and below, as in her case, was MGUS. She was followed up and about 8 years after the electrophoresis, developed severe back pain and it was obvious that she had multiple myeloma with many lytic lesions and was treated with cyclophosphamide (there being only this and melphalan to choose from at this time). She had some benefit; the protein decreased a little, but the disease progressed, and she died about 18 months later.

**MM:** So now we know the whole story about this MGUS patient. Let me move to a more personal question. Can you say a few words about the contribution of your family during your career? I met with your older son when you traveled to Paris together.

**RK:** I met my wife Charlene who was a nurse at the hospital that I interned in. She had had 2 years of college and then entered the 3-year nurses training program in which she obtained her Bachelor’s degree. When she finished her training, she came to Rochester as a nurse. In fact, she was the first radioisotope nurse at the Mayo Clinic. We married a year later. It was in Alaska that we had our first child, John, the one that you met. He became a high school teacher, teaching sciences, and he developed courses on the solar system and meteorology. He continued teaching in the same Minneapolis suburban high school. He is now retired and is in Australia visiting friends.

**MM:** So, you couldn’t attract your son to medicine?

**RK:** No, he was interested in the sciences though and we thought it best to let him choose his career. We have all seen people who have gone into medicine because they were pushed into it by a parent or grandparent. Our second child, Mary, graduated in Environmental Sciences and we asked what are you going to do with that? To which she replied, “I don’t know but I like it.” She married one of her classmates who earned a PhD in soil science and worked for a large research company, Battelle, in Washington state. Mary got a job in a laboratory with a professor in soil chemistry. After a year, he asked if she would like to get a Master’s degree and arranged for her to take the required classes. She did a project on growing alfalfa and the effect of adding phosphates. She proved that phosphates were necessary for the best crops. When pregnant with her first son, they moved to the Tri-Cities of Washington where her husband worked in his scientific field. She had three children so she stayed at home to look after them. At the age of 49, she told us that she’d like to be a nurse, so she took the training but declined getting a Master’s degree as she already had Bachelor’s and Master’s degrees. She went to work in an orthopedic hospital and became an expert in nursing patients after knee and shoulder surgeries. She retired about 10 years later as her children were then married and she’s now very busy with grandchildren.

Our third child Barbara became a pediatric nurse and then ran the teenage pregnancy program in Hennepin County, Minneapolis for a number of years. She is now in Public Health and responsible for the health of the Hennepin County high school students.

Our youngest child Jean got a Bachelor’s degree in Criminal Justice and Women’s Studies. She then studied law and has been a Public Defender in Hennepin County for the last 25 years. I believe that children should find their own way and do want they want to do, which is good advice for everyone.

**MM:** What would be your advice to the younger generation of doctors who want to be involved in myeloma and the gammopathies?

**RK:** I would begin by telling them to see all of the myeloma and amyloid patients in their practice—I’m talking about internists with an interest in multiple myeloma. When leaving here, for example, I encourage them to continue in the field. If they have gone to a university and are on faculty, I encouraged them to work with other persons in the field and develop the area that they find interesting and satisfying. If you are not vitally interested in what you are doing, it is going to be work. For me, it was fun, long days many times, but well worth it.

**MM:** This is fantastic to hear. It’s about passion! Is there anything you would have done differently in hindsight?

**RK:** Not really, I have enjoyed each phase, although it was frustrating to treat acute leukemia in the 1960s. I’m a persistent person and kept going. I’ve seen MGUS continue for 40 years without any change. The monoclonal gammopathies have shown me that 1% of those patients will progress each year to multiple myeloma or a related disorder. This has been borne out with continued experience. Ten percent of patients with smoldering multiple myeloma progress during the first year and then they decrease after that and ultimately after 10–15 years, progress at a rate of 1–2% per year. It all fits so beautifully, I think. I want to emphasize that I have seen patients virtually every day until I retired from clinical practice at the age of 72. I then worked in the Special Protein Laboratory that I had founded 40 years prior, for 5 years and then my colleague Jerry Katzmann who had been with me for over 20 years, took over and I was pleased that he did so. He is now retired, and David Murray is the leader and introduced the new area of mass spectrometry for the diagnosis of monoclonal gammopathies, so it is no longer electrophoresis with that familiar spike!

**MM:** This is really amazing Bob—your attention to detail. A couple of years ago, in one of his editorials Vincent Rajkumar highlighted that one of your main characteristics was your attention to detail, as well as your vision, your integrity, and your mentorship, you are a role model for all of us.

I have one last question—do you remember when we first met? In 1993, when you visited Montpelier and I was a medical student, you wanted to visit the old books at the Medical School because it is amongst the oldest Medical Schools in Europe.

**RK:** I certainly do. I particularly enjoy visiting libraries and one I remember had so many publications from the sixteenth and seventeenth centuries, they were commonplace. Here at the Mayo Clinic library, they would have been kept in the correct humidity and temperature. I would just like to add that I have enjoyed travelling immensely and I’m just completing my memoir and it has a special last chapter that tells of visits to various places. Places like Easter Island where I went with my son who had scheduled a whole week on this small island only 7 × 15 miles in size, but he was right to allow such a time as we were busy every day exploring the various sites. The Galápagos Islands, the Amazon jungles, Antarctica, Russia, Saudi Arabia, and a lot of very different places that I’ve been privileged to visit. I would always take a couple of weeks’ vacation each year just to travel to these out of the way places. We also have visited virtually all of our National Parks and have probably been to Yellowstone a dozen times. We are all “travelers” in the family.

**MM:** Thank you so much Bob, you are an international treasure for all of us and a living legend. We are always very grateful whether we be patient, physician, or health care professional for your countless contributions to patient care, to the field of multiple myeloma and gammopathy, to medical education, to research, and to mentorship. Thank you, it has been a true pleasure having this discussion with you and I hope that you have enjoyed it as much as I have learned from you today.

**RK:** Thank you very much Mohamad, I greatly appreciate the opportunity to speak, and sometimes, when you get me going, I fail to stop! This has been a very enjoyable interview and I hope that others like it as well. If there are any questions, I would be more than happy to attempt to answer them.

MM: Thank you, please stay safe and keep well.

